# Navigating the latent phase of labour: women’s experiences within structural constraints – a qualitative study from Germany

**DOI:** 10.1186/s12884-026-09382-w

**Published:** 2026-06-03

**Authors:** Caro Jeltsch, Sabine Rivière, Oda von Rahden

**Affiliations:** 1 Department of Technology and Health for People, Jade University of Applied Sciences, Oldenburg/Wilhelmshaven/Elsfleth, Germany; 2 Department of Obstetrics, Lüneburg Hospital, Lüneburg, Germany

**Keywords:** Latent phase, Midwifery care, Qualitative study, Social determinants, Equality

## Abstract

**Background:**

The latent phase of labour plays an important role in shaping women’s overall birth experiences. Limited professional support during this early, and often ambiguous, period can increase anxiety and undermine confidence. However, existing research infrequently examines how social and structural factors interact to influence women’s access to support during the latent phase of labour. Therefore, the aim of this study was to identify the informational and support needs of women in different contextual circumstances.

**Methods:**

This qualitative study explored how intersectional factors shape women’s experiences and access to care during their latent phase in labour in Germany. Twenty-two women, who had given birth in hospitals following a spontaneous onset of labour, participated in semi-structured interviews. Data were analyzed using qualitative content analysis according to Kuckartz, informed by participatory health-research principles.

**Results:**

Women described their preparations for the latent phase in diverse and individual ways, often characterized by uncertainty regarding the onset and progression of labour. Rather than referring to discrete stages, participants reported embodied sensations and decision-making processes unfolding as a continuum. Access to information and professional support during this period was shaped by structural factors, such as the regional availability of midwives, transport options, and language barriers. On-call and home-based midwifery services were perceived as highly reassuring, but remained accessible to only a few participants. In hospital settings, women sometimes reported inconsistent communication, limited reassurance, and uncertainty during the latent phase of labour.

**Conclusions:**

Experiences of the latent phase were shaped not only by individual factors but also by social and structural conditions. Strengthening equity in maternity care requires addressing financial, geographical, and linguistic barriers, to ensure that midwife-led, woman-centered support during latent labour becomes accessible to all women.

**Supplementary Information:**

The online version contains supplementary material available at 10.1186/s12884-026-09382-w.

## Background

Childbirth is a vulnerable and formative life event, with potential long-term consequences for maternal and infant health and wellbeing [1, 2]. Achieving positive birth experiences is therefore a central public health goal across Europe, where approximately five million children are born each year [3]. In line with WHO recommendations, a positive childbirth experience is defined as one in which women feel safe, respected, supported, and involved in decision-making, even when medical interventions are required [4]. The latent phase, which is part of the first stage of labour, plays a decisive role in shaping women’s overall perception of birth [5–7]. A lack of professional support at this stage can heighten anxiety and undermine women’s confidence [5]. Recent synthesis research highlighted that women’s care needs during early labour are primarily related to reassurance, information, and support in coping with uncertainty [8]. However, the German S3 guideline *Vaginal Birth at Term*, in line with other international guidelines such as those from NICE and the WHO, recommends hospital admission only once active labour has begun, as early admission is associated with higher intervention rates [4, 9, 10]. Thus, many women report feeling under-supported when sent home during the latent phase, or when admitted to hospital units lacking midwifery care [11, 12]. This discrepancy between women’s needs on the one hand, and clinical recommendations on the other, underscores the importance of understanding how women navigate the latent phase under current system conditions.

While a growing body of research has explored latent labour in terms of clinical management, care pathways, and supportive interventions, less attention has been paid to the intersection of social and structural factors with women’s experiential needs during the latent phase. Internationally, several innovative concepts for supporting women during latent labour have been developed, including dedicated early-labour support spaces, structured coping and decision-support tools, and mobility-focused frameworks [13–15]. Such initiatives are, however, often limited to specific regions or depend on additional funding [13–16], potentially restricting access to professional guidance during the latent phase.

According to the World Health Organization, the “social determinants of health” encompass the conditions in which people are born, grow, live, work, and age, including their access to resources and services [17]. These determinants, such as ethnicity, gender, occupation, and income, are widely recognized as shaping access to healthcare and support across the perinatal period [17]. In the context of latent labour, however, the intersection of social and structural factors with women’s experiential support needs remains insufficiently explored in existing research. This oversight limits our understanding of the complexities surrounding care access during latent labour, and highlights the need for further investigation into the ways in which these intersecting elements affect the delivery and quality of support provided to women in this context. Building on the WHO framework on social determinants of health [17] and informed by an intersectional perspective [18], this study explores how women’s experiences during the latent phase of labour are shaped through the interaction of social, biographical, and structural conditions. In line with intersectionality-based approaches, the study does not assume that women’s experiences can be adequately understood through isolated socio-demographic categories alone. Rather, attention is directed towards how multiple contextual factors — such as living conditions, previous experiences, social support, language-related challenges, or access to care — interact with institutional structures and organisational conditions of maternity services in shaping opportunities for support during latent labour. Following participatory health research principles [19, 20], the study therefore prioritised narrative accounts of lived experience over rigid demographic classification in order to capture the contextual complexity of women’s care trajectories.

Hence, this study aimed to explore how social and structural contextual conditions shape women’s information and support needs during the latent phase of labour. The objective was to ensure the highest possible level of participation among the target group. The study design was therefore informed by the theoretical framework of participatory health research, and builds on evidence that social determinants increasingly shape health opportunities in Western European societies [20–22].

## Methods

### Design

To explore women’s experiences of the latent phase of labour, a qualitative descriptive study design informed by participatory health research (PHR) principles was adopted. Data were generated through semi-structured expert interviews, following the approach described by Meuser and Nagel [23], focusing on participants’ experiential and context-specific knowledge. The analytical framework was guided by qualitative content analysis according to Kuckartz [24], allowing for a systematic combination of deductive and inductive category development. All participants provided written, informed consent. Audio recordings and pseudonymised transcripts were stored securely according to the EU GDPR. Ethical approval was granted by the Ethics Committee of the University of Oldenburg (file no. 2024-049). Reporting follows the COREQ checklist [25].

### Sampling and selection of participants

The initial target was to conduct 10–15 interviews with women who had given birth. A purposive sampling approach informed by principles of theoretical variation sampling was applied to capture a broad range of latent-phase care pathways and contextual experiences in Germany. This included women who initially remained at home before seeking hospital care, as well as women who planned a non-hospital birth but were admitted during the latent phase. Planned home births that proceeded entirely outside hospital care were excluded, as in these cases the latent phase is continuously monitored by a midwife and does not involve institutional assessment or decision-making within hospital structures. However, planned out-of-hospital births that resulted in hospital admission during the latent phase were included, provided that the latent phase was confirmed upon admission. Inclusion of these cases made it possible to explore how experiences of the latent phase unfolded within the hospital setting, including situations involving transfer between care contexts. The analytical focus remained on women’s experiences once hospital-based assessment and decision-making became relevant, regardless of their initial birth plan. Further inclusion criteria were a singleton pregnancy in cephalic presentation, spontaneous onset of labour, and a hospital birth following that onset. The time interval between birth and interview had to be within the previous two years, and the postpartum period had to be completed at the time of the interview. Both primiparous and multiparous women were included, as previous birth experiences may influence perceptions of the latent phase. The mode of birth was intentionally varied to explore whether overall birth experience influenced reflections on the latent phase. Sampling aimed to include variation in care pathways, support structures, and residential settings rather than predefined socio-demographic quotas. Recruitment was conducted through midwifery networks, family-support organisations, and the association Mother Hood e.V. across several German federal states (Lower Saxony, Schleswig-Holstein, Hamburg, and Bremen). Multiple recruitment channels were used to maximise accessibility and to include women across a range of life situations and care contexts. Within these organisations, staff and coordinators acted as gatekeepers by distributing study information and flyers to potentially eligible women, including women receiving social or family support services. Gatekeepers did not select participants or have access to interview data. All interested women contacted the researcher (CJ) directly and were screened for eligibility according to the inclusion criteria. Participation was voluntary and independent of any care relationship with the recruiting organisations. To reduce participation barriers, interviews were offered in German or English, and study materials were written in accessible language. According to the study protocol, sufficient proficiency in German or English was required to ensure informed consent and meaningful participation, as no professional interpretation services were available within the research team. In one case, a participant with limited confidence in actively speaking German, but sufficient understanding of the language, participated with the support of her husband, who provided informal oral translation of her responses from Arabic into German at her request. The participant had contacted the research team herself and was able to understand the interview questions directly. To enhance data accuracy and participant validation, the interview was transcribed, back-translated, and reviewed by the participant, who confirmed the content. Nevertheless, the use of informal translation may have influenced phrasing and interpretation and should therefore be considered a methodological limitation. At the same time, this pragmatic approach was chosen in line with participatory and low-threshold research principles in order to reduce exclusion of women with language-related barriers from participation in maternity care research. In accordance with participatory and low-threshold research principles [26, 27], no direct questions were asked about income, migration background, or other highly sensitive socio-demographic attributes. Instead, contextual information was derived from narrative accounts and systematically documented in analytic memos. This included contextual circumstances participants themselves described as relevant or challenging during pregnancy, birth, or access to care. While this approach limits formal socio-economic categorization, it aligns with the participatory focus of the study. Transferability was supported through purposive sampling for variation in life contexts, care pathways, and residential settings (urban, suburban, rural), as well as by providing a transparent description of contextual characteristics in the supplementary material.

### Data collection

The invitation materials and study information included a brief, accessible explanation of the latent phase of labour to ensure a shared understanding of the study focus prior to participation. The interview guide [supplementary material 1] was developed by two midwives (CJ and SR) based on an exploratory literature review, and finalized in a research workshop with experienced qualitative researchers. Feedback was obtained from the parents’ organization *Mother Hood e.V*., which pre-tested the relevance of key questions for current practice. The guide addressed women’s experiences of the latent phase, decision-making around hospital transfer, the roles of companions and professionals, and women’s sense of security during this period. Participants were also asked how well informed they felt before and during labour, and to suggest improvements for future latent-phase care. At the beginning of each interview, participants were explicitly asked whether they would like further clarification of the latent phase, to ensure a shared understanding before data collection began. While the interview guide referred to the onset of labour as an entry point into the topic, participants were encouraged to describe their experiences in their own terms and were not restricted to stage-based definitions.

Between 17 June 2024 and 15 October 2024, twenty-four interviews were conducted either in person or by telephone, with durations ranging from 28 to 65 min. Two interviews were excluded because the inclusion criteria were not fully met (preterm birth and stillbirth), resulting in a final sample of twenty-two women. Overall, due to the high level of interest in participating, the planned number of interviews was exceeded. One interview required translation from Arabic to German and vice versa. At the participant’s request, her husband carried out the oral translation. The transcript was back-translated, and then reviewed and confirmed by the participant. Despite targeted recruitment efforts, women facing language-related, social, or structural barriers remained underrepresented in the sample.

The initial target was to conduct 10–15 interviews with women who had given birth, guided by considerations of information power as proposed by Malterud et al. [28], whereby sample size in qualitative research depends on the specificity of the study aim, the quality of dialogue, and the richness of the data. As recruitment yielded a higher-than-expected level of interest in participation, interviews were continued to capture a broader range of experiences across different life contexts and care pathways. In particular, the recruitment of women from socially or structurally disadvantaged groups proved challenging, and additional interviews were conducted to enhance variation in perspectives rather than for reasons of convenience. Data collection and analysis proceeded iteratively. Recruitment was concluded once sufficient thematic depth and diversity of perspectives regarding experiences of the latent phase had been achieved and no substantially new thematic dimensions emerged in the ongoing analysis.

The interviewer (CJ) was a practicing midwife familiar with the topic. All interviews were audio-recorded, transcribed verbatim [29], and pseudonymised by removing identifying information (CJ and SR). The key linking participants’ names and interview codes was stored separately from the transcripts in a secure, password-protected file. To improve readability while maintaining authenticity, transcripts were linguistically refined [30]. Participants received their transcripts for communicative validation. Quotations used in this manuscript were translated into English. Initial translations of the quotations were generated using the translation software DeepL (DeepL SE, Cologne, Germany) and subsequently reviewed, adapted, and contextually refined by the first author in close discussion with the research team to preserve semantic meaning, tone, and idiomatic expression. Particular attention was paid to maintaining the authenticity of participants’ voices while ensuring readability in English. A native English-speaking editor with experience in academic writing reviewed the final translations. Original German quotations and their English translations are provided in Supplementary Material 2 to enhance transparency.

### Reflexivity and researcher characteristics

CJ, who conducted all interviews, is an academically trained midwife and qualitative researcher working in clinical maternity care. SR is also an academically trained midwife working in a clinical setting and was involved in data analysis and interpretation, but did not conduct interviews. Both researchers have professional expertise in maternity care, and SR has prior academic experience in qualitative research. Given the interviewer’s professional background, reflexivity was explicitly addressed throughout the research process. At the beginning of each interview, participants were informed that CJ was acting in the role of a researcher rather than a care provider, and that no professional advice, evaluation, or clinical judgement would be offered. This clarification aimed to reduce role-related expectations and minimize the perception of a hierarchical clinical relationship. At the same time, the shared professional familiarity with maternity care may have facilitated rapport and participants’ willingness to share detailed experiences. The researchers were aware that their professional commitment to woman-centred and midwifery-led care, as well as their engagement with current maternity care policy discussions, could shape interpretative perspectives, particularly regarding structural barriers and support needs during the latent phase [31]. To address this, analytic decisions were continuously reflected upon through reflexive memos and regular team discussions, with attention to alternative interpretations and to grounding findings closely in participants’ narratives rather than normative care models. To further enhance reflexivity and mitigate potential bias, semi-structured interview guides were used, reflexive memos were documented after each interview, and emerging interpretations were regularly discussed within the research team to critically reflect on positionality, assumptions, and the potential influence of the researchers’ clinical backgrounds on data collection and analysis.

### Analysis

Data were analyzed using qualitative content analysis following Kuckartz [24], supported by MAXQDA 2022©. This is a systematic and rule-guided approach that allows the combination of deductive and inductive category development. The method aims to structure and reduce textual data, while maintaining contextual meaning and ensuring transparency of analytical decisions. In a first step, preliminary main categories were deductively derived from the interview guide and the study’s research questions. All transcripts were then independently reviewed by two researchers (CJ and SR), and relevant text passages were assigned to these initial categories. In a second step, categories were inductively refined through close reading and constant comparison across cases: categories were modified, differentiated, merged, or discarded, and additional subcategories were developed based on emerging themes within the data. This iterative process resulted in a hierarchically structured category system comprising five main categories and twelve subcategories, which formed the framework for the final coding process. To enhance rigour, the dual-control principle was applied during coding, and analytic memos were used to document interpretive decisions and category revisions. This was achieved using a consensus-based dual-control approach. During the final coding phase, both researchers (CJ and SR) jointly reviewed transcripts and assigned text passages to categories. In cases of disagreement, discrepancies were resolved through discussion, drawing on the category definitions and the contextual meaning of the data. This process also led to iterative refinement and clarification of category definitions where ambiguities became apparent. To further support credibility, selected excerpts and the finalized category system were discussed in a research colloquium, where additional researchers reviewed the allocation of anchor quotes to categories and provided critical feedback on their conceptual fit. Reflexivity was understood as an ongoing process of critically reflecting on positionality, interpretation, and researcher influence throughout data collection and analysis [31] and was maintained through regular team discussions. The final category system and anchor quotations are presented in Table 1.

## Results

A total of 22 women participated in the study, including 12 primiparous and 10 multiparous participants. Most births were spontaneous vaginal births (*n* = 19), alongside two caesarean sections and one vacuum-assisted birth. Participants represented a range of latent phase care pathways, including hospital-based care, home-based latent phase care followed by hospital admission, interrupted planned out-of-hospital births, and cases with continuous midwifery support in hospital. In line with the study’s focus on social determinants, the sample reflected diverse social and contextual circumstances relevant to accessing support during the latent phase. Participants lived in urban (*n* = 11), suburban (*n* = 5), and rural (*n* = 6) areas, with varying travel times to hospital (from < 10 min to > 30 min), indicating differences in geographic accessibility to maternity services. Women also described a range of contextual circumstances that they experienced as challenging during pregnancy and birth, including language-related barriers, previous negative birth experiences, limited social support, or difficult living situations. These narrative accounts provided contextual insight into differing support resources, care conditions, and structural circumstances shaping experiences of the latent phase. A detailed overview of participant characteristics is provided in Supplementary Material 3.

Although interviews focused on the latent phase of labour, participants rarely described their experiences using stage-based terminology, but rather as a continuous and fluid process. Nevertheless, the interview guide focused exclusively on questions related to the latent phase, and participants had been informed of this focus in advance. As the study relied on retrospective interviews, the exact clinical transition from latent to active labour could not be objectively verified. No access to medical records was obtained, and women were not continuously examined during labour. The identification of the latent phase was therefore based on participants’ retrospective accounts in relation to remembered clinical events (e.g., hospital admission, discharge, or confirmation of active labour). The analysis identified five main themes describing women’s experiences of the latent phase of labour: (1) preparation for the latent phase, (2) navigating the latent phase without continuous professional support, (3) change of setting, (4) the latent phase in hospital, and (5) feeling safe and supported while navigating the latent phase. These themes capture how women prepared for early labour, managed uncertainty and decision-making at home, experienced the transition to hospital care, and perceived safety and support across different care contexts. Across themes, women’s experiences were shaped by the interplay of personal resources, professional support, and organisational conditions of care. The following sections present these themes in detail, illustrated by participant quotations. The main categories are presented in the following section, while Table 1 provides a complete overview of all main- and subcategories. Following qualitative content analysis according to Kuckartz, selected anchor quotations are used to illustrate each category. To enhance readability, the narrative presentation focuses on the main categories, while subcategories are reflected within the text and illustrated through selected quotations rather than being presented as separate subheadings. The original German quotations are available upon reasonable request to the corresponding author.

**Table 1:** Category system

Main category and definition	Subcategory	Anchor quote and Reference
Preparation for the latent phase: The upcoming birth is the focus of this main code, which contains descriptions of measures or thoughts related to it. These may be organizational, emotional, or physical.		
	*Thoughts on the onset of labour*: This subcode contains descriptions of ideas and thoughts about the onset of labour and how to organize everyday life around it. It also contains descriptions of a care concept for the latent phase, provided it is eligible.	English quote: *“[…] I tried to read the signs*,* because my second birth happened really fast*,* and this time I wanted to make sure I got in the car—got to the hospital—in time. Last time we just didn’t make it. So I kept checking the whole time*,* really paying attention to what the signs were. Like*,* are these real contractions now*,* or just the early ones? And I was actually pretty nervous*,* because I was scared I’d miss that moment again.” (I11*,* phr. 4)*
	Sources of information and support: This subcode contains a description of the sources of information and opportunities for exchange that were used in preparation for birth.	English quote: *“Yes*,* we did a childbirth prep class. In person*,* you know*,* on site […] That actually got me pretty well prepared for the birth itself*,* at least in theory [laughs]. And on top of that I did an online course—‘The Peaceful Birth.’ Yes*,* that… that also prepared me pretty well*,* especially for the early phase. […] I actually felt really well prepared.” (I6*,* phr. 8)*
Navigating the latent phase without continuous professional support:This main code contains the description of the course of the latent phase spent outside the hospital setting.		
	*Activity* This subcode covers statements about the activities that the woman in labour and her birth companions undertake during the latent phase, with the aim of promoting labour, relieving pain, and passing the time until it is time to move to the delivery room.	English quote: *“So then we went inside and tried*,* like*,* a thousand different positions at home*,* and at some point I just said*,* ‘Nope*,* this just isn’t working.’ So we went upstairs and lay down in bed*,* and that was a total disaster. I said*,* ‘No*,* if it keeps going like this*,* I won’t make it through the birth.’ The pain was just intense. Yeah*,* and then at some point we figured out that standing was better*,* that being in the shower for hours was better […].” (I12*,* phr. 9)*
	*Experiencing the onset of labour:* This subcode provides descriptions of the cognitive and emotional experiences associated with the latent phase within a home setting.	English quote: *“In that moment I was like*,* ‘Great*,* why did I even do all that prep if none of what people tell you is actually true?’ I always thought timing contractions meant you time every contraction*,* not only once they hit a certain intensity. So I was just standing there thinking*,* ‘What is even going on right now?’”(I8*,* phr. 16)*
	*Contact with maternity care providers*: This subcode provides a description of interactions with healthcare professionals, such as midwives and gynaecologists, during the latent phase.	English quote: *“And then I kind of woke up*,* and it was like… the contractions were gone again. But they kept coming back. And because I was really unsure*,* I remember texting my midwife and asking if I could come in so she could check whether they were real contractions. And [name of midwife] basically said*,* if I’m still wondering whether they’re real*,* then they probably aren’t yet — that I’d definitely know when they were the real thing.” (I11*,* phr. 12)*
Change of setting The main code includes descriptions of the transition from home to hospital settings.		
	*Decision-making on transfer*: This subcode contains statements on individual reasons for changing location, or for or against inpatient admission after transfer. It includes a description of the criteria used to select the location during the latent phase, as well as the expectations associated with this location. This includes transfers from home in cases of planned out-of-hospital births.	English quote: *“That was really the moment when I realized it was draining my energy really fast*,* because I didn’t have those breaks anymore to catch my breath and recharge. And I felt like I’d kind of used up everything I’d learned*,* so to speak. That’s when I wanted to talk to my midwife face-to-face and see what options there were.” (I10*,* phr. 68)*
	*Expieriencing the transfer:* The subcode provides a description of the physical and emotional experience in the moments leading up to and during the journey to hospital, as well as reflections on the timing of admission.	English quote: *“For me it already felt kind of strange when we left the house*,* because as soon as the apartment door closed behind us it was like*,* okay*,* when we come back*,* we’ll be a little family. That was really emotional for me. […] So there was this kind of uneasy feeling. […] At home I still felt totally comfortable. But at the clinic I suddenly had a bit of respect for the whole situation*,* because I didn’t know what to expect or what the next few hours would bring. And I also didn’t know when things would actually really start.” (I2*,* phr. 43)*
The latent phase in the hospital:The main code contains descriptions of the time spent in hospital during the latent phase.		
	*Admission procedure and diagnosis* This subcode contains a description of the hospital admission process, as well as the experience associated with diagnosing the ‘latent phase’.	English quote: *“And the midwife who checked us in was a bit reserved at first. […] And yeah*,* at some point she came back and basically told me that nothing had really happened yet. Like*,* my cervix just hadn’t opened any further. And the CTG didn’t show*,* you know*,* any strong contractions either. She also told me I should expect a lot more before things would really get going. And honestly*,* I found that really insensitive in that moment.” (I6*,* phr. 36)*
	Care resources and decision making: This subcode describes the care situation in hospital and the decision-making processes for obstetric measures during the latent phase.	English quote: *“[…] and then it kind of felt like*,* ‘Okay*,* I’m in the hospital now*,* something big is happening*,* and it’s not really in my hands anymore.’ Even though they really did try to keep me informed and include me and ask about my preferences. But frequently it just overwhelmed me. It was hard when they’d ask me things right as I was having a contraction and I was supposed to make decisions while[…]. At that point I didn’t really feel connected to myself anymore. I didn’t know*,* like*,* ‘What do I need right now? What actually helps me?’ And I shifted more and more into*,* ‘I just have to rely on whatever they tell me.’” (I15*,* phr. 34)*
Feeling safe and supported while navigating the latent phase:This main code provides a description of the factors influencing physical and emotional well-being during the latent phase.		
	*Shared dynamics between woman and birth companion* This subcode provides a description of the influence that the woman giving birth and her companions have on their experience during the latent phase.	English quote: *“My partner is a really relaxed person*,* and that really helped me — just knowing he was optimistic. Like*,* ‘We’ve got this*,* you can do it*,*’ and he kept reminding me of that. That was really important to me*,* and I think I also told him how important it was that if needed*,* he could step out and get someone. So that he could kind of communicate on my behalf*,* because sometimes that can be tricky in the hospital if no one else is around.” (I3*,* phr. 55)*
	Setting: This subcode provides descriptions of how local settings influence the experience of the latent phase.	English quote: *“[…] what I had here at home was this feeling of*,* ‘I can’t really let go because I’m scared I’ll wake [older child’s name]’ — everything else was fine. I could basically just do my thing. I was at home and both of them [partner and the midwife] were there. I didn’t have that in the hospital. I could be loud there*,* sure*,* but I didn’t know how things worked*,* what the routines were. And because of that I still couldn’t let go.” (I5*,* phr. 46)*
	*Counselling and care experiences* This subcode provides descriptions of how professional support and counselling influence the experience of the latent phase.	English quote: *“I was supposed to move around for half an hour*,* just walk up and down. And then during the next check-in she came back — or actually a doctor came with her — and he told me I was still in the latent phase and could just go for another walk outside. And I was like*,* ‘Dude*,* have you even looked at my file? I’ve been pacing around here for 30 hours and you’re talking to me about the latent phase?’ I was so mad at him. […] In that moment it felt like a slap in the face. I really felt like he was treating me like I didn’t know anything. […]” (I5*,* phr. 20)*

*I* Interview, *phr.* paragraph

### Preparation for the latent phase

Women described preparing for the latent phase in highly individual ways, shaped by their living conditions, expectations, and available sources of information. Preparation involved organizational, emotional, and physical aspects, and was often accompanied by a mix of anticipation and uncertainty, particularly regarding the recognition of the onset of real labour. Several women expressed concerns about misinterpreting bodily signals or arriving at the hospital too early, sometimes drawing on previous birth experiences.

*“With my first [child]*,* I went to the hospital once for nothing*,* because I was having those really strong false contractions — and at that time*,* I didn’t really know what real contractions felt like. […] I remember having something similar with my second birth — my son was actually born in the car*,* right in front of the hospital. Just before that*,* I remember thinking*,* ‘Oh great*,* I’m making such a fuss*,* they’ll all come running out with the doctors*,* and it’s probably not even time yet!’” (I11*,* phr. 28)*.

To manage uncertainty and gain a sense of control, some women developed personal preparation strategies, such as writing a birth plan, visiting the planned birth location, or arranging on-call midwife support. Midwives and other mothers were described as central sources of information about the latent phase. In-person birth preparation classes were valued for the opportunity to exchange experiences, although access was sometimes limited due to inadequate availability.

*“[…] [midwife] talked to us loads during the pregnancy about the latent phase — what to expect and all that — so my partner was really on board as well […]. But it really does help when someone else says it too [laughs]*,* So*,* […]*,* I think we were actually pretty well sorted when it came to things you can use to get through the latent phase.” (I4*,* phr. 8)*.

Online resources, podcasts, and social media were also mentioned by several participants. While these formats were easily accessible, some women reported that such resources reinforced negative expectations or heightened anxiety, rather than providing reassurance.

### Navigating the latent phase without continuous professional support

Experiences of the latent phase at home were characterized by varying perceptions of pain, progress, and support needs. Some women managed early contractions independently for extended periods, while others described a growing need for emotional or practical support as the intensity increased. This was reflected in statements such as

*“The problem was that I’d let [partner’s name] sleep […] But then I realized I really needed his support — and by that point I was in constant pain.” (I1*,* phr. 48)*.

Through phone contact with professionals, several women sought reassurance and attempted to clarify whether labour had begun. Approximately half of the participants described contacting a known midwife during this phase, primarily to receive professional confirmation and emotional reassurance.

*“[…] I remember that I texted my midwife and asked if I could come in to have it checked*,* because I wasn’t sure whether these were real contractions. […] She said that if I still had to ask*,* then they probably weren’t real yet.” (I11*,* phr. 12)*.

Awareness of limited delivery-room capacity also shaped decision-making, leading some women to inquire about availability before leaving home.

*“Up here in [big city] in general*,* before you set off*,* […] you should call ahead to see if the delivery room/if they have capacity.[…] And then we called and she said*,* ‘No’ […].” (I21*,* phr. 22)*.

Overall, women did not describe childbirth as a sequence of clearly demarcated stages. Instead, experiences of uncertainty, reassurance-seeking, and changing support needs unfolded along a continuum, which women retrospectively associated with the latent phase of labour.

### Change of setting

Decisions to leave home were influenced by multiple factors, including recommendations from midwives, physical symptoms, and uncertainty about the progress of labour. Some women chose to go to hospital proactively to avoid arriving too late, while others described being encouraged by their partners. Due to increasing pain and restricted mobility, transfers were sometimes experienced as physically demanding and emotionally challenging, particularly as contractions intensified during the journey.

*“[…] I had felt strong at the beginning*,* but that was completely gone. I was exhausted*,* and the contractions became increasingly intense. During the ambulance ride*,* it was already difficult to even have a conversation between contractions […] it was all very overwhelming.” (I15*,* phr. 10)*.

In some cases, the choice of hospital was limited due to capacity constraints, which evoked frustration and loss of composure. Some women described a sense of relief upon arrival at the hospital, anticipating increased support and medical reassurance. Some women who faced longer travel distances, limited transport options, or lacked midwifery support described additional stress and uncertainty related to the timing of transfer, highlighting geographic disparities in access to care.

*“Exactly*,* and then we couldn’t go there [chosen hospital]. But they said*,* ‘We’ll call the neighbouring hospital.’ […] They were also full*,* so we couldn’t go there either. Then another hospital was recommended to us*,* in another town. […] That’s when I lost my composure*,* because that would be a different town.” (I6*,* phr. 36)*.

### The latent phase in the hospital

Admission to hospital during the latent phase was sometimes described as disruptive or unpleasant. Routine procedures such as cardiotocography restricted mobility, and were perceived as delaying access to pain relief. Some women experienced inconsistent communication between hospital staff and community midwives, which intensified feelings of distress.

*“I walked in and said*,* ‘I need something for the pain. I just can’t do this anymore.’ The car ride had already been awful — you can’t exactly sit however you want in there. And then the midwife was like*,* ‘No*,* no*,* no*,* first we’ll do a CTG [cardiotocography] and check again.’ But my homebirth midwife had already listened to the baby’s heartbeat and done an exam — she’d checked everything*,* the baby was fine*,* I could feel her moving. Everything was okay*,* and I just completely lost it. (I1*,* phr. 54)*

Some women reported receiving interventions during this period, such as analgesia or labour augmentation. In addition to these clinical aspects, organisational features of care — particularly staff availability and continuity — were described as shaping women’s experiences. As accounts were based on subjective experiences rather than clinical assessments, the exact timing of interventions in relation to labour stages could not always be determined with certainty.

Experiences of decision-making varied: while some women felt informed and involved, others perceived a loss of control or felt pressure to consent despite ambivalence. Perceived limitations in staff availability and continuity further influenced women’s experiences. In particular, a lack of continuous presence was associated with increased uncertainty and a sense of responsibility to actively seek support.

*“It wasn’t like someone was constantly there*,* making you feel like they were watching you closely. It was more like they would check in whenever they could. You kind of have to make sure yourself that you actually get a bit of support.” (I3*,* phr. 55)*.

### Feeling safe and supported while navigating the latent phase

Women’s accounts of the latent phase often emphasized the importance of feeling safe, supported, and emotionally contained while navigating an uncertain and evolving process. This sense of comfort was shaped through the interplay of personal resources, the presence and role of companions, the physical environment, and the quality of professional care and counselling. The following section presents these influences as they emerged from women’s narratives. Central to this sense of safety was the presence of a trusted companion and confidence in one’s own body. Adequate knowledge of physiological processes helped reduce uncertainty for both women and their companions, whereas limited understanding often increased anxiety. The emotional state of the accompanying person was perceived as directly influencing the woman’s ability to remain calm and focused. In this context, companions were sometimes described as potential advocates who could communicate with obstetric staff when women felt unable to do so themselves. In this context, companions were described not only as sources of emotional support, but also as important intermediaries in situations where continuous professional presence was perceived as limited.

*“My partner is a really relaxed person*,* and that really helped me — just knowing he was optimistic. […] That was really important to me*,* and I think I also told him how important it was that if needed*,* he could step out and get someone. So that he could kind of communicate on my behalf*,* because sometimes that can be tricky in the hospital if no one else is around.” (I3*,* phr. 55)*.

Beyond external support, trust in one’s own body and in the natural process of birth was described as an important internal resource. Some women felt confident in listening to their bodily sensations, while others — particularly primiparous women or those with previous negative experiences — found this more difficult and described uncertainty about interpreting latent labour signals.

*“Listening to yourself — I think that’s the most important thing. But I don’t know if you can really do that with your first child. I couldn’t*,* and I didn’t.” (I22*,* phr. 60)*.

In addition to interpersonal and internal resources, the physical environment played an important role in shaping women’s comfort and autonomy. Home settings were associated with familiarity, freedom of movement, and the ability to respond intuitively to bodily needs. In contrast, hospital environments were sometimes perceived as restrictive, particularly when monitoring procedures limited mobility. These accounts highlight a tension between familiarity and control on the one hand, and institutional constraints on the other, which shaped women’s sense of autonomy during the latent phase. In this context, the physical environment is considered in terms of how it shapes women’s sense of comfort, autonomy, and safety, rather than as a structural feature of care provision.

*“I could tell nothing was really moving forward. I would have liked to walk around or something*,* but that just didn’t really work. […] I was stuck on the CTG the whole time*,* which was super annoying*,* because I didn’t really want that in the first place.” (I1*,* phr. 60)*.

Within the clinical setting, some women attempted to recreate a sense of familiarity by bringing personal items from home. While this could enhance comfort, institutional routines and spatial constraints continued to limit independence.

*“So*,* what was actually pretty nice was that I took my nursing pillow with me in the ambulance*,* […] that made me really comfortable. It was like*,* you know*,* having a little bit of home with me*,* something familiar around. […]” (I15*,* phr. 54)*.

Hospital settings were also described as ambivalent spaces, simultaneously associated with vulnerability and reassurance. Sharing rooms, or being subject to visiting restrictions, sometimes intensified feelings of exposure, while for others, the immediate availability of medical assistance provided a strong sense of safety.

*“And then*,* when I got to the ward*,* […] I just knew that if anything happened*,* help would be there right away. […] That was really my main thought — that I was safe*,* basically.” (I17*,* phr. 41)*.

Together, these findings illustrate how the physical environment was experienced not only as a setting of care, but as an active factor shaping women’s sense of safety, autonomy, and vulnerability. Beyond these contextual and relational aspects, experiences of professional care and counselling further influenced whether women felt supported or unsettled during the latent phase. They were sometimes linked to staffing levels, continuity, and communication styles. Feeling seen, taken seriously, and guided through decision-making processes fostered trust and emotional security. Conversely, inconsistent messages, or frequent changes in caregivers, were described as distressing and undermining confidence.

*“[…] You really give a lot of trust up front to the people who are there with you and guiding you through it — I think it’s such a unique situation. You just hope you end up with the right people*,* you know? “(I19*,* phr. 28)*.

*“What was really hard for me was having different people check my cervix — three or maybe even four of them. […] I mean*,* it makes sense that different people might assess things differently*,* but in such a vulnerable situation*,* that’s just […] not great. […] Like*,* when the night shift tells me*,* ‘We’ll start an oxytocin drip now*,*’ and then the next person comes in and says*,* ‘You should get some sleep*,*’ I just find that really unhelpful.” (I5*,* phr. 24)*.

Communication barriers further complicated experiences of care. Language differences and insensitive communication styles could contribute to misunderstandings and feelings of disrespect, resulting in women disengaging from interactions altogether. In one case, a participant chose to be represented by her partner during the interview due to language barriers. The following quote reflects her experience as conveyed by her husband:

*“She [woman interviewed] said she asked her [staff] to speak more slowly*,* but the midwife — or whoever it was — didn’t speak slower*,* just louder*,* and with these big eyes and everything. You’d think [name of the woman] was blind and deaf or something. She was like*,* “What’s that supposed to mean?” And at that point*,* she just stopped trying to understand because she’d had enough of that kind of behaviour. (I9 (husband)*,* phr. 260)*

Several women also described distress arising from conflicting information or perceived inattentiveness, particularly during phone consultations in the latent phase. In this context, the absence of a continuous support person was not primarily framed as a structural issue, but as affecting women’s sense of safety and confidence in their own perceptions. In retrospect, some participants described how the presence of a doula or another continuous support person could have provided reassurance and advocacy, particularly in situations where their own bodily experiences did not align with professional advice.

*“If someone had been there*,* I would have said*,* ‘Can you please just check how far along I am?’ because there was such a discrepancy between how I felt and how she [midwife on the phone] felt.” (I8*,* phr. 38)*.

## Discussion

This study highlights that women’s experiences of the latent phase of labour are shaped by a complex interplay of personal and structural factors. Access to continuous midwifery support varied depending on regional service availability, awareness of care options, and early planning. Participants described several barriers, including limited availability of home-based midwifery care, limited continuity of care and availability of support in hospital settings, language barriers, and capacity constraints in maternity units. These structural conditions influenced not only access to care but also decision-making processes, such as when and how to transfer to hospital.

Women’s experiences of latent labour were shaped not only by individual coping strategies, but also by broader organisational and social conditions.In addition, this study provides insight into how women experience the latent phase of labour as an uncertain, relational, and socially mediated process rather than a clearly bounded clinical stage. Women did not describe the latent phase in stage-based biomedical terms, but as a fluid continuum characterized by ambiguity, reassurance-seeking, and ongoing sense-making. The findings suggest that the latent phase is experienced less as a physiological phase alone, and more as a period of interpretative work, in which women continuously assess bodily sensations, negotiate uncertainty, and decide when to seek support. This highlights a fundamental tension between biomedical stage-based models of labour and women’s lived, process-oriented experiences. The need for reassurance, validation, and emotional containment emerged as a central experiential theme across settings, which is consistent with recent synthesis research highlighting reassurance and guidance as key care needs during early labour [8].The findings, particularly those related to *change of setting* and *the latent phase in hospital*, demonstrate that access to information and professional support—particularly during the latent phase—is largely shaped by regional health-care service availability and institutional conditions. The restrictions may more strongly affect women with limited transport options, language barriers, or limited health literacy, than significantly more privileged persons [17].

### Social and structural influences on access to care

The results related to *preparation for the latent phase*, *change of setting*, and *the latent phase in hospital* indicate that women’s access to support was shaped by structural conditions rather than individual preferences alone, a pattern also described in recent research on early labour care needs and service organization [8, 32].While these findings are situated within the German maternity care context [33], similar challenges have been described in other high-income health systems, where access to continuous support during latent labour depends on service organization, workforce availability, and care pathways [34–37]. Although care during the latent phase is often framed as guided by women’s preferences, participants’ options were in practice constrained by the local availability of midwifery services, transport options, and hospital capacity. In Germany, midwives can provide home-based care during the latent phase only if they hold obstetric insurance, which substantially limits service provision [16]. However, the underlying issue of limited continuity of care in latent labour is not unique to Germany, but reflects broader structural tensions between community-based and hospital-based maternity care models. In addition, spatial and logistical factors—such as transport options, travel distance, and familiarity with available maternity units—further shaped women’s experiences and decision-making processes during the latent phase. These findings resonate with international discussions on the centralization of obstetric services and increasing travel distances to maternity units, which have been reported across several European and other high-income settings [34–37]. From an experiential perspective, the latent phase may represent a period of increased uncertainty and perceived vulnerability, in which gaps in service coordination and accessibility become particularly visible. As reflected in participants’ accounts, this was often associated with uncertainty, a need for reassurance, and challenges in interpreting bodily signals.

### Individual strategies within structural constraints

Despite these constraints, women described actively developing individual strategies to cope with uncertainty during the latent phase. Approximately half of the participants reported seeking contact with a known midwife during this period, valuing professional reassurance and support in assessing labour progress. However, access to such continuity depended on financial resources, regional service structures, and awareness of available options. Women without access to continuous midwifery care more frequently reported uncertainty and, in some cases, a perceived loss of control — particularly in situations where support was limited or inconsistent and communication was experienced as fragmented. While participants did not attribute these experiences to specific causes, such patterns have been associated in the literature with organisational constraints, including staffing pressures, in maternity care settings [8, 38]. The difficulty in retrospectively demarcating the latent phase further underscores the tension between biomedical stage-based models of labour and women’s lived experiences.

### Towards equitable and woman-centered care

Participants consistently emphasized the importance of reassurance, continuity, and being taken seriously during labor. However, some hesitated to seek help for fear of being perceived as a burden on services. Addressing this ambivalence requires environments that normalize support during the latent phase, and value communication and relational continuity. Out-of-hospital or hybrid models that bridge home and hospital—such as early labour lounges or structured ambulatory care—may offer a more flexible and equitable response to women’s needs during this phase [13–16]. The evidence and feasibility of these could be reviewed for Germany and other countries. Evidence from participatory and co-design approaches show that such concepts can improve both outcomes and satisfaction [39]. Ensuring a safe and timely transfer also requires attention to transport options, particularly in rural areas.

### Implications for equity in latent-phase care

The findings underscore the importance of relational continuity, timely reassurance, and accessible support during the latent phase, rather than focusing solely on clinical progression or admission thresholds. Although women facing more pronounced language-related, social, or structural barriers were only partly represented in the sample, the findings suggest that substantial challenges in accessing support during the latent phase were already evident across a wide range of care situations and life contexts. It can therefore be assumed that restricted access to midwifery-led care, limited transport options, language barriers, or maternity unit closures pose even greater challenges for women facing social or structural disadvantage. Ensuring equitable access to midwife-led care is a matter of social justice and public health. The results support international calls to strengthen midwife-led, woman-centered care concepts that explicitly include structured support during the latent phase [38].

Achieving this requires sustainable support structures for accessible midwife-led care, improved cooperation between community- and hospital-based providers, and targeted information strategies. Addressing social and structural barriers should be an integral component of maternity service planning. Strengthening support during the latent phase has the potential to improve women’s experiences by reducing uncertainty, enhancing reassurance, and facilitating more appropriate timing of admission. This may also contribute to avoiding unnecessary early admissions and, indirectly, to a more efficient use of hospital resources. However, as this qualitative study did not assess clinical outcomes, any potential association between enhanced latent-phase support and obstetric interventions should be interpreted with caution. Future mixed-methods and quantitative research is needed to examine whether structured out-of-hospital or hybrid latent-phase care models are associated with intervention rates, such as induction or caesarean section, and with workload distribution in maternity services. If centralization of obstetric care continues, complementary care concepts alongside hospital-based care will become increasingly important. Policies should therefore prioritize reducing socioeconomic and geographic inequalities to ensure that all women—regardless of income, education, or place of residence—can benefit from continuous and person-centered support during the latent phase of labour.

### Limitations and strengths

This study has several limitations that should be considered when interpreting the findings. Despite efforts to recruit participants across diverse care settings and life situations, women facing more pronounced language-related, social, or structural barriers were only partly represented in the sample. This may limit transferability to populations experiencing greater challenges in accessing maternity care and support during the latent phase. As a qualitative study, the aim was analytical rather than statistical generalization, focusing on in-depth understanding and transferability to comparable maternity care contexts.

In addition, no standardized socio-demographic variables (e.g., education, employment, or income) were systematically collected. Consequently, assessments of social advantage or vulnerability are based on narrative contextual information rather than objective socio-economic indicators, which limits the formal evaluation of the sample’s socio-structural composition. Professional interpretation services were also not systematically available; although one interview included participant-approved informal translation, this may have reduced the inclusion of women with lower language proficiency and reflects structural language barriers relevant both to research participation and maternity care access.

Participation was voluntary and may have introduced self-selection bias. Furthermore, as interviews were conducted up to two years after birth, recall bias cannot be excluded, and accounts may reflect retrospective sense-making. The latent phase was identified based on women’s narratives rather than verified clinical data, as no medical records were accessed and the transition to active labour could not be precisely delineated. The findings therefore represent subjective constructions of the early phase of labour rather than strict definitions of the clinical stage.

Despite these limitations, the study offers rich, in-depth insights into an under-researched phase of childbirth and highlights the interplay between individual experiences, social circumstances, and structural conditions. Methodological rigour was strengthened through systematic qualitative analysis, dual coding, communicative validation, and adherence to COREQ reporting standards [25].

## Conclusion

This study highlights that women’s experiences of the latent phase of labour are shaped not only by individual needs and coping strategies, but also by structural conditions influencing access to support. Limited continuity of care, geographic barriers, language-related challenges, and restricted availability of midwifery services affected women’s sense of safety and reassurance during this period. Strengthening equitable access to midwife-led and woman-centred support during the latent phase may help improve women’s experiences and reduce inequalities in maternity care.

## Supplementary Information

Supplementary Material 1.

Supplementary Material 2.

Supplementary Material 3.

## Data Availability

The datasets generated and analysed during the current study are not publicly available due to privacy- and data-protection restrictions, but are available from the corresponding author on reasonable request.
