# Supplementary material for: Navigating the latent phase of labour: women’s experiences within structural constraints – a qualitative study from Germany
Source: BMC Pregnancy Childbirth. 2026 Jun 3;26:601. doi: 10.1186/s12884-026-09382-w (PMC13231696; doi:10.1186/s12884-026-09382-w)
Supplement: Supplementary file 1 — Supplementary Material 1. [file 12884_2026_9382_MOESM1_ESM.docx]

**Interview guide**

## Before the interview begins

- Introduction of the interviewer
- Ask whether the participant prefers a more informal or a more formal form of address
- Explanation of the research project and data protection procedures
- Obtaining verbal and written consent for participation in the interview

**Introduction:**

Thank you very much for taking the time to talk to me about the birth of your child. I understand that this can be a very sensitive topic. If at any point during the conversation you feel uncomfortable, please let me know right away. You are free to skip any question, ask for a break, or end the interview at any time. There are no “right” or “wrong” answers to the questions I will ask. I am interested in hearing about your own experiences and your personal perspective. I will not evaluate or comment on your answers in any way. Everything we discuss will remain confidential. The interview will only be shared with others in anonymized form, so that no conclusions can be drawn about your identity.

Do you have any questions before we start? And are you ready to begin the interview?

**Introductory question:**

To begin with, could you briefly describe your life situation during your pregnancy and in the first few months after your child was born?

**Follow-up prompt:**
Were there any aspects of your life during pregnancy that caused you concern?
For example: your living situation, financial situation, mobility, or relationship.

**Topic 1: Preparing for childbirth**

As the pregnancy goes on, many questions about the birth of the baby often come up. How did you prepare for labor?

| **Question** | **Follow-up prompts** | **Check/ Keywords** |
| --- | --- | --- |
| When did you start thinking about the upcoming birth? | What preparations have you made for the onset of labor?  How did you imagine the ideal onset of labor? | preparation  antenatal care |
| How did you feel during your pregnancy when you thought about the onset of labor? | Have your feelings about the onset of labor changed during your pregnancy? | feelings |
| What information did you have about the onset of labor? | What information did you have about what to do when labor starts? | information  support |
| With whom have you discussed the upcoming birth? | Who supported you in preparing for the birth? | antenatal care  gynaecologist  midwife  social environment |

**Topic 2: Decision criteria for choosing the place of birth**

Where did you give birth to your child, and what were your reasons for choosing that particular place?

| **Question** | **Follow-up prompts** | **Check/ Keywords** |
| --- | --- | --- |
| Why this particular birthplace and no other? | What factors led to the choice of this birthplace, if there was a choice? | quality criteria |
| Did you visit the birthplace before the birth? | What did you like (or dislike) about your first visit? | information  assessment |
| What were your expectations of this birthplace? | Were expectations met? | quality of care |
| In retrospect, would you choose the same place of birth again? | Why (not)? |  |

**Topic 3: subjective onset of labor/latent phase**

Now, think back to the moment your child was born. Imagine the first signs that labour has started and your baby is on its way. Describe the start of your child's birth to me.

| **Question** | **Follow-up prompts** | **Check/ Keywords** |
| --- | --- | --- |
| How did you know that labor was starting? | How is it different from before? | signs  feelings |
| How did you feel during this time? | What were your thoughts? | emotions  thoughts |
| Where were you when the contractions first started, before you went to hospital? | Why were you there? | location |
| What did you do during that time? | Did you have any idea what would help you during this time? | activity  calmness  information |
| Who was with you? | Why was this person there? | social environment  partner  midwife  doula |
| What physical or emotional changes have you noticed in yourself over time? | How did the changes feel? | safety |

**Topic 4: Need for security**

In Germany, pregnancies are usually monitored very closely through regular check-ups, and many aspects are planned in advance. At the same time, childbirth itself is difficult to plan. What was your experience of this? What was important to you in order to feel safe and well cared for at the start of labor?

| **Question** | **Follow-up prompts** | **Check/ Keywords** |
| --- | --- | --- |
| Did you feel safe during the onset of labor? | What made you feel (un)safe?  What did you miss when you felt unsafe? | safety |
| What was important to you in order to feel well cared for? | How do you imagine the ideal onset of labor? | surroundings  accompaniment |
| How did you imagine the care you would receive during the onset of labor? | What did you expect from the care provided at the onset of labor? | counselling  care |
| Did you feel well cared for during the early phase of labor? | How did you feel about the care you received? | care |

**Topic 5: Decision criteria for admission**

What made you decide to leave for hospital at that particular moment?

| **Question** | **Follow-up prompts** | **Check/ Keywords** |
| --- | --- | --- |
| What changes had there been in the hours before? | How did these changes feel to you?  *If there were no changes:* How did you feel during this time? | changes  emotions |
| What expectations or wishes did you have at that time regarding the care you would receive? | How would you like things to change as a result of the relocation? | Reasons for transfer |
| How long did you think about setting off before you actually did so? | What considerations led to the decision to leave? | criteria  instinct  need for security |
| Which mode of transport did you use to get to the hospital? | Why did you choose this mode of transport?  How long did it take you to get there? | mobility  social environment  need for security |
| What was your experience of getting to the hospital? | How did you feel about it?  What did you think? |  |

**Topic 6: Experience at the hospital**

Please tell me about what happened after you arrived at the hospital.

| **Question** | **Follow-up prompts** | **Check/ Keywords** |
| --- | --- | --- |
| What happened after arriving at the hospital? | What diagnosis/prognosis did you receive?  What measures were taken after arrival? | diagnosis  information  measures |
| What were your needs and wishes at that time? | In which situations were these (not) fulfilled? | needs  wishes |
| How did you feel after moving? | What physical or emotional changes did you notice after moving? | emotions  changes |
| Who made the decision about the further course of care? | What information about beneficial measures was available to you at that time? | cecision making  hospital care |
| How did you experience the care provided by midwives and doctors at that time? | Which expectations and wishes regarding the change of location could be fulfilled by the staff? | staff |

**Topic 7: Role of a birth companion**

Please tell me whether and how you were supported by a birth companion during the onset of labor.

| **Question** | **Follow-up prompts** | **Check/ Keywords** |
| --- | --- | --- |
| Who accompanied you? | What were your reasons for choosing your companion?  *If you did not have a companion:* Why not? | companion  properties |
| What did you expect from your companion? | What had you discussed before the birth? | wishes  expectations  accompaniment |
| How was your companion able to support you upon arrival at the hospital? | How could your companion have supported you (even) better? | support |
| Was your partner involved in the decision-making process? | To what extent do you think your companion should be involved in the decision-making process? | decision making |
| Did you discuss the onset of labor with your companion afterwards? | What do you know about your companion's memories of the onset of labor? | debriefing |

**Topic 8: Wishes and ideas for better care**

Looking back now on the beginning of your child’s birth, how could it have been ideal for you?

| **Question** | **Follow-up prompts** | **Check/ Keywords** |
| --- | --- | --- |
| What thoughts come to mind when you recall the onset of labor? | What would you do differently in hindsight?  Did anything about the experience surprise you? | review |
| In retrospect, what kind of support would have been helpful to you during the initial stage of labor? | What additional support would have been helpful to you at the onset of labor?  Would you have liked to have had a special point of contact for the beginning of labor? | support |
| What (additional) information would you have liked to have had? | In hindsight, who would you have liked to talk to (more) about the upcoming birth? | information |
| What advice would you give to another woman about the onset of labor? |  | support |

Thank you very much for trusting me and for speaking so openly about your experience of giving birth to your child. Is there anything else that is important to you and that we have not yet talked about?
