# Supplementary material for: Navigating the latent phase of labour: women’s experiences within structural constraints – a qualitative study from Germany
Source: BMC Pregnancy Childbirth. 2026 Jun 3;26:601. doi: 10.1186/s12884-026-09382-w (PMC13231696; doi:10.1186/s12884-026-09382-w)
Supplement: Supplementary file 2 — Supplementary Material 2. [file 12884_2026_9382_MOESM2_ESM.docx]

**Table 2: Quotations**

| **Reference** | **English translation** (used in manuscript) | **Original German quotation** |
| --- | --- | --- |
| I11,  phr. 28 | *“With my first, I went to the hospital once for nothing, because I was having those really painful Braxton Hicks contractions — and at that time, I didn’t really know what real contractions felt like. […] I remember having something similar with my second birth — my son was actually born in the car, right in front of the hospital. Just before that, I remember thinking, ‘Oh great, I’m making such a fuss, they’ll all come running out with the doctors, and it’s probably not even time yet!’”* | *“Beim ersten war ich einmal umsonst da, weil ich halt auch diese schmerzvollen Senkwehen hatte und damals ja auch noch gar nicht wusste, was jetzt wirklich Wehen sind. […] ich weiß auch noch, dass ich das bei der zweiten Geburt auch hatte, also mein Sohn ist im Auto gekommen vor dem Krankenhaus und als ich/ ich weiß noch, kurz vor der, also bevor er gekommen ist, dachte ich noch so: ‘Boah jetzt mache ich hier so eine Welle, nachher kommen die jetzt raus gerannt mit den Ärzten und es ist noch gar nicht so weit.’* |
| I4,  phr 8 | *“And yeah, [name of the home birth midwife] talked to us* loads *during the pregnancy about the latent phase — what to expect and all that — so my partner was really on board as well. And of course I then went over all of that with him again myself, like,* so *many times. But it really does help when someone else says it too [laughs], when it comes from another person [laughs]. So yeah, I think we were actually pretty well sorted when it came to things you can use to get through the latent phase and all that — at least in our heads [laughs].”* | *“Und, genau, [Name der Hausgeburtshebamme] hat uns in der Schwangerschaft ganz viel über die Latenzphase erzählt, was zu erwarten ist, damit natürlich mein Freund auch mit im Boot ist. Dem habe ich natürlich ganz ganz viel noch erzählt. Aber es hilft natürlich auch oft, wenn noch jemand von außen [lacht] nochmal was sagt, jemand anderes [lacht]. Genau, deswegen waren wir da, glaube ich, auch, was Hilfsmittel für die Latenzphase und so angeht, total gut aufgestellt. Rein kognitiv [lacht].”* |
| I1,  phr. 48 | *“The problem was that I’d let [partner’s name] sleep, because at first I felt like, okay, I can handle this on my own. But then I realized I really needed his support — and by that point I was in constant pain.”* | *“Also da war dann das Problem [...], dass ich [Name des Partners] dann noch habe schlafen lassen, weil ich gemerkt habe, okay, ich mache das alleine. Und dann habe ich aber gemerkt, jetzt brauche ich ihn irgendwie als Unterstützung und ich habe [....] Dauerschmerzen gehabt […].* |
| I11,  phr. 12 | *“[…] I remember that I texted my midwife and asked if I could come in to have it checked, because I wasn’t sure whether these were real contractions. […] She said that if I still had to ask, then they probably weren’t real yet.” (I11, phr. 12)* | *„Und da ich irgendwie ziemlich unsicher war, ich weiß auch noch, dass ich meiner Hebamme dann geschrieben habe und habe gefragt, ob ich vorbeikommen könnte, um das zu checken, ob das echte Wehen sind. Und [Name der Beleghebamme] hatte aber da gesagt, wenn ich mich schon frage, ob es echte Wehen sind, dann sind es bestimmt noch keine, ich würde es wissen, wenn es echte Wehen sind.“* |
| I21,  phr. 22 | *Up here in [big city] in general, before you set off, and it's nothing drastic or anything, you should call ahead to see if the delivery room/if they have capacity. Or if you can call back in an hour or something. And then we called and she said, ‘No’ […].”* | *Hier oben in [Großstadt] generell, bevor man losfährt und es ist nichts krasses irgendwie, soll man vorher anrufen, ob der Kreißsaal/ ob die Platz haben. Oder ob man in einer Stunde nochmal anrufen kann und so. Und dann haben wir angerufen und die meinte so: “Nee” […].* |
| I15,  Phr. 10 | *“[…] I had felt strong at the beginning, but that was completely gone. I was exhausted, and the contractions became increasingly intense. During the ambulance ride, it was already difficult to even have a conversation between contractions […] it was all very overwhelming.” (I15, phr. 10)* | *„Also ich war dann/ also ich hatte ja am Anfang noch dieses, dass ich mich so stark gefühlt habe und so, das war total weg. Also ich war richtig fertig, also die Wehen fingen dann auch an, richtig anstrengend zu sein, obwohl in der Intensität auch in der Häufigkeit klar wurde, das wird immer ein bisschen mehr, aber so dieses war nicht im Verhältnis zu wieviel schwerer es sich dann auf einmal angefühlt hat. So und ich war dann bei – ach, ich weiß ich gar nicht – ich hatte die auch getrackt, bei alle drei bis fünf Minuten irgendwie ne Wehe. Und habe dann gemerkt, so dieses im/ im RTW auch nochmal Gespräche führen und dann zwischendurch immer/ immer atmen müssen [lacht]. Also das war dann alles irgendwie schon anstrengend.“* |
| I6,  phr. 36 | *“Exactly, and then we couldn't go there. But they said, ‘We'll call the neighboring hospital.’ I knew from experience [...] from others that it was also a great hospital, in the same town. They were also full, so we couldn't go there either. Then another hospital was recommended to us, in another town. [...] That's when I lost my composure, because that would be a different town.”* | *„Genau und dann konnten wir dort nicht hin. Die haben aber gesagt: “Wir rufen im Nachbar-Krankenhaus an.” Ich wusste […] aus Erfahrung von anderen, dass das auch ein super Krankenhaus ist, gleiche Stadt. Die waren auch voll, da konnten wir auch nicht hin. Dann wurde uns noch ein anderes Krankenhaus empfohlen, in der anderen Stadt. […] Da habe ich dann erstmal die Contenance verloren, weil das ja dann eine andere Stadt wäre.“* |
| I1,  phr. 54 | *“I walked in and said, ‘I need something for the pain. I just can’t do this anymore.’ The car ride had already been awful — you can’t exactly sit however you want in there. And then the midwife was like, ‘No, no, no, first we’ll do a CTG and check again.’ But my homebirth midwife had already listened to the baby’s heartbeat and done an exam — she’d checked everything, the baby was fine, I could feel her moving. Everything was okay, and I just completely lost it. I thought, ‘This can’t be happening — now I have to lie down for another twenty minutes while they run the CTG again and check me once more before I get anything for the pain.’”* | *“[...] Und ich bin da rein und habe gesagt, ich brauche jetzt was gegen Schmerzen. Ich kann gerade einfach nicht mehr. Die Autofahrt war schon echt irgendwie schlimm. Da kannst du dich ja nicht irgendwie hinsetzen, wie du willst. Und dann meinte sie [Hebamme]: ‘Nee, nee, nee, wir schreiben jetzt erstmal CTG und tasten jetzt erst noch mal’ und [Name der Hausgeburtshebamme] hatte daheim Kindstöne abgehört, daheim einmal getastet, hatte quasi alles einmal erhoben, ihr [dem Kind] ging es gut, ich habe sie gespürt. Das war alles irgendwie fein und ich habe echt eine Krise bekommen, weil ich dachte, das kann doch nicht sein, dass ich mich jetzt nochmal 20 Minuten dahin lege, um noch mal ein CTG zu schreiben und dann noch mal zu tasten und dann erst was gegen die Schmerzen bekomme.”* |
| I3,  phr. 55 | *“ […]. It wasn’t like someone was constantly there, making you feel like they were watching you closely. It was more like they would check in whenever they could. You kind of have to make sure yourself that you actually get a bit of support.”* | *“ [...] also es war jetzt nicht so, dass da dauernd jemand war und man das Gefühl hatte, die, ne, gucken wirklich sehr genau auf einen. Eher so, die gucken halt so immer wieder, wenn es halt gerade ging. Man muss schon auch selber so ein bisschen dafür sorgen, dass halt, ja, dass man so ein bisschen Betreuung vielleicht auch kriegt.”* |
| I3,  phr. 55 | *“My partner is a really relaxed person, and that really helped me — just knowing he was optimistic. Like, ‘We’ve got this, you can do it,’ and he kept reminding me of that. That was really important to me, and I think I also told him how important it was that if needed, he could step out and get someone. So that he could kind of communicate on my behalf, because sometimes that can be tricky in the hospital if no one else is around.”* | *“[...] Der [Partner] ist auch eher ein sehr entspannter Mensch und dass mir das halt geholfen hat, dass er da einfach so optimistisch war. So, wir schaffen das und du schaffst das und dass er mir das noch mal so ganz stark vermittelt [...]. Und das war mir halt wichtig und ich hatte ihm, glaube ich, auch noch irgendwie gesagt, [...]dass mir das total wichtig ist, dass er dann halt sonst rausgeht und jemanden holt. Also dass er für mich sozusagen mit kommunizieren kann, weil das ja manchmal schon im Krankenhaus bisschen schwierig sein kann, wenn einfach gerade (...) keiner da ist.”* |
| I22,  phr. 60 | *“Yeah, and listening to yourself — I think that’s the most important thing. But I don’t know if you can really do that with your first child. I couldn’t, and I didn’t.”* | *“Ja und auf sich hören, ist glaube ich das Allerwichtigste. Aber ich weiß nicht, ob man das so kann beim ersten Kind. Ich hätte es nicht gekonnt und ich konnte es nicht.”* |
| I1,  phr. 60 | *“[…] I could tell nothing was really moving forward. I would have liked to walk around or something, but that just didn’t really work. [...] And that was, for me — I was stuck on the CTG the whole time, which was super annoying, because I didn’t really want that in the first place.”* | *“[…] Ich habe das ja auch gemerkt, dass nicht so richtig was vorwärts geht. Ich wäre auch gerne rumgelaufen oder so. Aber das hat halt irgendwie nicht richtig funktioniert. [...] Ja und das war für mich, also ich war auch dauerhaft am CTG, super nervig, weil ich das ja eigentlich auch nicht wollte.”* |
| *I15,*  *phr. 54* | *“So, what was actually pretty nice was that I took my nursing pillow with me in the ambulance, [...] that made me really comfortable. It was like, you know, having a little bit of home with me, something familiar around .[…].”* | *„Also was zum Beispiel ganz cool war, ich habe mein Stillkissen mitgenommen in den RTW quasi und war da gut/ gut gelagert und hatte quasi, ne, so, ne, nochmal was von zu Hause mit dabei, so, und was Vertrautes um mich, so. […].“* |
| I17,  phr. 41 | *“And then, when I got to the ward, […] I immediately felt really comfortable. I just knew that if anything happened, help would be there right away. [...] That was really my main thought — that I was safe, basically.”* | *“Und dann, als ich auf der Station ankam, […] habe ich mich dann auch sofort sehr wohlgefühlt und ich wusste einfach, wenn jetzt irgendwas ist, ist Hilfe sofort da. [...] Das war einfach so mein Gedanke, dass ich in Sicherheit bin, quasi.”* |
| I19,  phr. 28 | *“[…] You really give a lot of trust up front to the people who are there with you and guiding you through it — I think it’s such a unique situation. You just hope you end up with the right people, you know? Because for me, the whole birth really depends on that. […].”* | *“[…] Man gibt direkt ein großes [Vertrauen] in Vorschuss an die Personen, die sich da befinden und den Prozess begleiten, das ist, glaube ich, eine einmalige Situation. Man hofft einfach, dass man an die richtigen Personen kommt, ne. Weil damit geht und steht für mich die Geburt. [...]“* |
| I5,  phr. 24 | *“What was really hard for me was having different people check my cervix — three or maybe even four of them. [...] I mean, it makes sense that different people might assess things differently, but in such a vulnerable situation, that’s just […] not great. […] Like, when the night shift tells me, ‘We’ll start an oxytocin drip now,’ and then the next person comes in and says, ‘You should get some sleep,’ I just find that really unhelpful.”* | *„Also was sehr schwierig für mich war, waren diese verschiedenen Muttermundsbefunde durch drei bzw. vier verschiedene Personen.* *Es ist für mich logisch, dass bei verschiedenen Personen die Einschätzungen verschieden ausfallen können. Aber in so einer vulnerablen Situation, in der ich mich befunden habe, ist das, ja, (...) ungünstig. […] Ja, weiß ich nicht, wenn sie mir/ wenn die Nachtschicht mir sagt […] Wir machen jetzt einen Wehentropf und dann die neue Person kommt und sagt: “Du gehst jetzt schlafen”, dann finde ich das ungünstig.“* |
| I8,  phr. 38 | *If someone had been there, I would have said, ‘Can you please just check how far along I am?’ because there was such a discrepancy between how I felt and how she [midwife on the phone] felt. I don't think I would have argued then. So I think I would have asked if she could just check it. If someone had been there.* | *Wenn jemand da gewesen wäre, dann hätte ich gesagt: “Kann man bitte einfach checken, wie weit ich bin?”, weil das halt so eine Diskrepanz war, von dem, wie ich´s empfunden habe und wie sie es empfunden hat. Da hätte ich dann, glaube ich, nicht diskutiert. Also da hätte ich dann, glaube ich, gefragt, ob sie es vielleicht einfach mal checken kann. Wenn jetzt jemand da gewesen wäre.* |
| I5, phr. 24 | *“What was really hard for me was having different people check my cervix — three or maybe even four of them. [...] I mean, it makes sense that different people might assess things differently, but in such a vulnerable situation, that’s just […] not great. […] Like, when the night shift tells me, ‘We’ll start an oxytocin drip now,’ and then the next person comes in and says, ‘You should get some sleep,’ I just find that really unhelpful.”* | *Es ist für mich logisch, dass bei verschiedenen Personen die Einschätzungen verschieden ausfallen können. Aber in so einer vulnerablen Situation, in der ich mich befunden habe, ist das […] ungünstig. […] wenn die Nachtschicht mir sagt […]: „Wir machen jetzt einen Wehentropf.“ und dann die neue Person kommt und sagt: “Du gehst jetzt schlafen”, dann finde ich das ungünstig.“* |

I= Interview, phr.= paragraph
