# Supplementary material for: Navigating the latent phase of labour: women’s experiences within structural constraints – a qualitative study from Germany
Source: BMC Pregnancy Childbirth. 2026 Jun 3;26:601. doi: 10.1186/s12884-026-09382-w (PMC13231696; doi:10.1186/s12884-026-09382-w)
Supplement: Supplementary file 3 — Supplementary Material 3. [file 12884_2026_9382_MOESM3_ESM.docx]

**Table 3: Sample**

| **No.** | **Parity** | **Stress factors of living situation at birth (narrative)** | **Mode of birth** | **Previous mode of birth** | **Latent phase care** | **Residence (urban/ rural)** | **Transport vehicle for transfer** | **Travel time to hospital** |
| --- | --- | --- | --- | --- | --- | --- | --- | --- |
| **1** | 1 | full-time student;  Partner in training (works shifts) | spontaneous vaginal | / | home (interrupted home birth) | urban | car (from a friend) | 10 minutes |
| **2** | 1 | not mentioned | spontaneous vaginal | / | home-based latent phase care | urban | car | 5 minutes |
| **3** | 2 | not mentioned | spontaneous vaginal | Vacuum extraction with episiotomie and Kristeller´s maneuver | hospital | urban | taxi | ? |
| **4** | 1 | not mentioned | spontaneous vaginal | / | home (interrupted home birth) | urban | car | 20 minutes |
| **5** | 2 | partner in training;  no further family network available | spontaneous vaginal | caesarean section after labor induction | hospital (after home-based latent-phase care) | urban | car | < 5 minutes |
| **6** | 1 | not mentioned | caesarean section | / | hospital | rural | car | 15 minutes |
| **8** | 1 | just moved to another city; dependent on public transport | spontaneous vaginal | / | hospital  (attendant midwife) | rural | taxi | 40 minutes |
| **9** | 1 | language barrier | spontaneous vaginal | / | hospital | urban | car | 5 minutes |
| **10** | 1 | not mentioned | spontaneous vaginal | / | hospital  (attendant midwife) | suburb | car | 15 minutes |
| **11** | 3 | not mentioned | spontaneous vaginal | 1: spontaneous vaginal  2: spontaneous vaginal, precipitate delivery in the car | hospital  (attendant midwife) | suburb | car | ? |
| **12** | 1 | partner in training | spontaneous vaginal | / | hospital  (attendant midwife) | rural | car | 20 minutes |
| **13** | 2 | not mentioned | spontaneous vaginal | spontaneous vaginal  (very fast) | hospital | rural | car | 30 minutes |
| **14** | 2 | very exhausting pregnancy with many infections and a broken rib | spontaneous vaginal | spontaneous vaginal after augmentation with oxytocin | hospital | suburb | car | 20 minutes |
| **15** | 1 | single parent by choice;  child has two fathers | caesarean section | / | home (interrupted birth in birthing center with latent-phase care at home) | urban | ambulance | 35 minutes (not nearest hospital) |
| **16** | 2 | patchwork family;  negative birth experience;  breech presentation until due date | spontaneous vaginal | caesarean section | hospital | urban | car | 5 minutes |
| **17** | 2 | not mentioned | spontaneous vaginal | spontaneous vaginal with Epidural | hospital | rural | car | 45 minutes |
| **18** | 1 | not mentioned | spontaneous vaginal | / | hospital | rural | car | 30 minutes |
| **19** | 2 | dependent on public transport | spontaneous vaginal | vacuum extraction with Kristeller´s maneuver | hospital | urban | ambulance | ? |
| **20** | 2 | negative birth experience | spontaneous vaginal | spontaneous vaginal | hospital | suburb | car | 20 minutes |
| **21** | 1 | employer does not comply with maternity protection law (e.g. smoking in the rooms); severe fear of childbirth | vacuum extraction | / | hospital | suburb | car | 20-25 minutes |
| **22** | 2 | negative birth experience | spontaneous vaginal | spontaneous vaginal with manual placenta removal | hospital | rural | car | 40 minutes |
| **23** | 1 | fear of childbirth; initially planned elective caesarean section | spontaneous vaginal with manual placenta removal | / | hospital | urban | car | 10-15 minutes |
| **Total: 22** | 12 primiparous  10 multiparous |  | 19 spontaneous vaginal  1 vacuum extraction  2 caesarean sections |  | 2x home  1x birth center  2x hospital after home-based latent-phase care  4x attendant midwife (hospital)  13x hospital | urban: 11  suburb: 5  rural: 6 | car: 18  taxi: 2  ambulance: 2 | < 10 min: 5.  10-30 min.: 10  > 30 min.: 4  not known: 3 |
